# Effects of lipid extraction on nutritive composition of winged bean (*Psophocarpus tetragonolobus*), rubber seed (*Hevea brasiliensis*), and tropical almond (*Terminalia catappa*)

**DOI:** 10.14202/vetworld.2018.446-451

**Published:** 2018-04-10

**Authors:** Anuraga Jayanegara, Rakhmad P. Harahap, Richard F. Rozi, Nahrowi

**Affiliations:** Department of Nutrition and Feed Technology, Faculty of Animal Science, Bogor Agricultural University, Bogor 16680, Indonesia

**Keywords:** *Hevea brasiliensis*, *in vitro* rumen, lipid extraction, *Psophocarpus tetragonolobus*, *Terminalia catappa*

## Abstract

**Aim:**

This experiment aimed to evaluate the nutritive composition and *in vitro* rumen fermentability and digestibility of intact and lipid-extracted winged bean, rubber seed, and tropical almond.

**Materials and Methods:**

Soybean, winged bean, rubber seed, and tropical almond were subjected to lipid extraction and chemical composition determination. Lipid extraction was performed through solvent extraction by Soxhlet procedure. Non-extracted and extracted samples of these materials were evaluated for *in vitro* rumen fermentation and digestibility assay using rumen: Buffer mixture. Parameters measured were gas production kinetics, total volatile fatty acid (VFA) concentration, ammonia, *in vitro* dry matter (IVDMD) and *in vitro* organic matter digestibility (IVOMD). Data were analyzed by analysis of variance and Duncan’s multiple range test.

**Results:**

Soybean, winged bean, rubber seed, and tropical almond contained high amounts of ether extract, i.e., above 20% DM. Crude protein contents of soybean, winged bean, rubber seed, and tropical almond increased by 17.7, 4.7, 55.2, and 126.5% after lipid extraction, respectively. *In vitro* gas production of intact winged bean was the highest among other materials at various time point intervals (p<0.05), followed by soybean > rubber seed > tropical almond. Extraction of lipid increased *in vitro* gas production, total VFA concentration, IVDMD, and IVOMD of soybean, winged bean, rubber seed, and tropical almond (p<0.05). After lipid extraction, all feed materials had similar IVDMD and IVOMD values.

**Conclusion:**

Lipid extraction improved the nutritional quality of winged bean, rubber seed, and tropical almond.

## Introduction

Ruminants can convert high-fiber feed materials such as grasses and agricultural residues into nutritious foods for a human. This is possible due to the presence of symbiotic microorganisms (bacteria, anaerobic fungi, etc.) in the rumen of ruminants that produce various degrading enzymes including cellulolytic enzymes. However, feeding of grasses or agricultural residues is insufficient to supply adequate nutrients, particularly protein, for supporting optimal production of the animals [1]. Protein supplements such as oilseeds, legumes, agro-industrial by-products, and animal by-products are therefore often used to satisfy protein demand of ruminants [2-4]. Soybean and soybean meal (lipid extracted soybean) are common protein supplements used in animal production, both ruminant and monogastric animals, due to their excellent protein characteristics. Soybean contains a high amount of protein within the range of 35-52% dry matter (DM) [5]; its protein is easily digested and rich in essential amino acids such as lysine, threonine, and tryptophan [6]. Notwithstanding, exploration of unconventional protein supplements is needed to reduce dependency on soybean. This is of practical importance for farmers with limited ability to purchase commercial supplements or farmers with limited access to obtain soybean.

Winged bean (*Psophocarpus tetragonolobus*), rubber seed (*Hevea brasiliensis*), and tropical almond (*Terminalia catappa*) are protein-rich materials and grow abundantly in tropical regions. A number of previous studies have reported the protein contents of winged bean, rubber seed, and tropical almond, i.e., 40.3, 21.9, and 20.1% DM, respectively [7-9], and the values were generally lower in comparison to soybean except for winged bean. These materials also contain substantial amounts of lipids that may hamper rumen degradation and fermentation when present in excessive amount in the rumen, particularly due to negative effects of lipids on population and activity of cellulolytic microbes [10,11]. Lipids in oilseeds, therefore, need to be removed to prevent their negative effects on rumen microbes and fermentation. Such lipid removal would increase proportion of protein in the materials so that they are comparable or even contain higher protein than that of soybean meal and may enhance protein supply for the host animals. The extracted lipids may further be used for various industrial applications such as for pharmaceuticals, cosmetics, food and feed supplements, renewable energy, surfactants, detergents, and others [12,13].

The present study aimed to evaluate the nutritive values of winged bean, rubber seed, and tropical almond and their *in vitro* rumen fermentability and digestibility, before and after lipid extraction. Soybean as a common protein supplement, both full-fat and lipid-extracted soybean, was used as a reference material for evaluating the nutritive values of winged bean, rubber seed, and tropical almond.

## Materials and Methods

### Ethical approval

Rumen content, i.e., liquid part and solid particle used in the present experiment was obtained from two fistulated Ongole crossbreed cattle before morning feeding at Biotechnology Research Center, Indonesian Institute of Sciences, Cibinong Bogor, Indonesia. The cattle were cared for according to the Animal Welfare Guidelines of Indonesian Institute of Sciences. Approval of the experiment was granted from the Faculty of Animal Science, Bogor Agricultural University, Indonesia.

### Extraction procedure and chemical composition determination

An amount of 1 kg soybean, winged bean, rubber seed, and tropical almond each was collected from field experimental station of Bogor Agricultural University, Indonesia. Each sample was dried in an oven at 60°C for 24 h. Dried sample was ground using a hammer mill with a screen size of 1 mm. Ground sample of soybean, winged bean, rubber seed, and tropical almond was subjected to lipid extraction procedure and chemical composition determination. Lipid extraction was performed through solvent extraction by Soxhlet procedure. Approximately 40 g of each sample was placed in a porous cellulose thimble. The thimble containing sample was then placed in an extraction chamber that was suspended above a flask containing solvent (petroleum ether) and below a condenser. Extraction was conducted at boiling point for 6 h to result residue with low-lipid content. Both non-extracted and extracted soybean, winged bean, rubber seed, and tropical almond were subjected to chemical composition determination, i.e., ether extract (EE), crude protein (CP), and crude fiber (CF) contents. These analyses were performed according to the AOAC [14] in duplicate for each material.

### In vitro fermentation and digestibility assay

The non-extracted and extracted samples of soybean, winged bean, rubber seed, and tropical almond were evaluated for *in vitro* rumen fermentation and digestibility assay by following the procedure of Theodorou *et al*. [15]. An amount of 0.75 g sample was mixed with 75 ml filtered rumen content and McDougall’s buffer (rumen content: buffer 1:4 v/v) in a 125 ml serum bottle. Incubation medium and serum bottles were continuously flushed with carbon dioxide gas to ensure anaerobic condition before the incubation started. Serum bottles were immediately closed with butyl rubber stoppers and aluminum crimp seals to start the incubation. For determination of *in vitro* gas production kinetics, two serum bottles per feed material were incubated for 72 h in a water bath at a constant temperature of 39°C. Gas production was vented and recorded at 2, 4, 6, 8, 12, 24, 36, 48, and 72 h after incubation using a syringe equipped with a needle. For determination of fermentation products, i.e., total volatile fatty acid (VFA) and ammonia, and *in vitro* digestibility, another two bottles per feed material were incubated in two stages [16] for 2×24 h. After the first 24-h incubation, supernatant was taken out by centrifugation for total VFA and ammonia measurements as described in Jayanegara *et al*. [17]. In the second 24-h incubation, the residue was further incubated with 75-ml pepsin-HCl 0.2 N for determination of *in vitro* DM digestibility (IVDMD) and *in vitro* organic matter digestibility (IVOMD). Initial amounts of DM and OM were subtracted with their corresponding DM and OM residues to obtain IVDMD and IVOMD, respectively. *In vitro* incubations were conducted in three runs.

### Data analysis

Data were analyzed by general linear model procedure with factorial arrangement 4×2. The first factor was different feed materials (soybean, winged bean, rubber seed, and tropical almond), whereas the second factor was lipid extraction (non-extracted and extracted). Allocation of treatments into experimental units followed a randomized complete block design, in which different *in vitro* runs served as the block due to variation of rumen microbial population and activity within each sampling time. Outlier was detected and removed from the dataset when Z score was lower than −2 or higher than 2. For comparison among different treatment means, Duncan’s multiple range test was employed provided that the parameter tested was significant at p<0.05. Data analysis was performed using IBM SPSS Statistics software version 20.

## Results

Soybean, winged bean, rubber seed, and tropical almond contained high amounts of EE, i.e., above 20% DM (Table-1). Lipid extraction of these materials using petroleum ether resulted in low EE contents, that is, below 1% DM. Winged bean contained CP higher than that of soybean, whereas CP contents of rubber seed and tropical almond were similar. The CP contents of soybean, winged bean, rubber seed, and tropical almond increased by 17.7, 4.7, 55.2, and 126.5% after lipid extraction, respectively. All the feed materials had CF lower than 10% DM. Lipid extraction of soybean, winged bean, rubber seed, and tropical almond resulted in increasing CF by 32.3, 79.2, 8.1, and 34.7%, respectively, as compared to their unextracted forms.

**Table-1:** Chemical composition of soybean, winged bean, rubber seed, and tropical almond either without or with lipid extraction.

Feedstuff	Lipid extraction	EE (% DM)	CP (% DM)	CF (% DM)
Soybean	NE	35.6	33.4	9.6
	E	0.60	39.3	12.7
Winged bean	NE	20.4	42.8	7.2
	E	0.20	44.8	12.9
Rubber seed	NE	37.6	21.2	6.2
	E	0.30	32.9	6.7
Tropical almond	NE	52.1	21.9	7.2
	E	0.36	49.6	9.7

CF=Crude fiber, CP=Crude protein, DM=Dry matter, EE=Ether extract, E=Extracted, NE=Non-extracted

**Figure-1:**
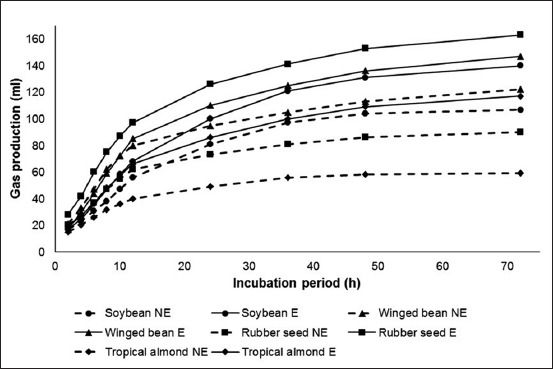
*In vitro* gas production profile of soybean, winged bean, rubber seed, and tropical almond either without non-extracted or with lipid extraction (E) at various incubation period.

*In vitro* gas production of intact winged bean was the highest among other materials at various time point intervals (p<0.05), followed by soybean > rubber seed > tropical almond (Figure-1). Extraction of lipid increased *in vitro* gas production of soybean, winged bean, rubber seed, and tropical almond (p<0.05). After the extraction, the highest *in vitro* gas production was observed in the incubation of rubber seed, followed by winged bean > soybean > tropical almond.

Among the intact feed materials, the highest total VFA concentration was obtained in winged bean incubation, whereas the parameter was indifferent between soybean, rubber seed, and tropical almond (Table-2). Lipid extraction increased total VFA concentration of all feed materials (p<0.05). With regard to ruminal ammonia concentration, incubation of all feed materials resulted in high ammonia concentrations, i.e., above 25 mmol/l. Variation of this parameter was rather high among different feedstuffs and lipid extraction. Similar IVDMD and IVOMD were observed among intact soybean, winged bean, and rubber seed; they possessed higher IVDMD and IVOMD than those of tropical almond (p<0.05). Lipid extraction increased IVDMD and IVOMD of soybean, winged bean, rubber seed, and tropical almond as compared to their intact forms (p<0.05). After lipid extraction, all feed materials had similar IVDMD and IVOMD values.

**Table-2:** *In vitro* rumen fermentation and digestibility of soybean, winged bean, rubber seed, and tropical almond either without or with lipid extraction.

Feedstuff	Lipid extraction	VFA (mmol/l)	NH_3_ (mmol/l)	IVDMD (%)	IVOMD (%)
Soybean	NE	51.7±10.1^a^	42.1±8.8^bc^	73.1±3.2^b^	72.4±5.1^b^
	E	67.4±8.8^cd^	41.0±3.2^bc^	89.3±0.8^c^	85.6±3.1^c^
Winged bean	NE	61.2±8.5^bc^	36.1±10.9^abc^	76.3±3.8^b^	74.6±6.5^b^
	E	79.5±1.5^e^	46.1±6.6^c^	86.5±1.1^c^	86.4±1.9^c^
Rubber seed	NE	55.2±8.9^ab^	25.3±7.3^a^	72.7±4.9^b^	71.5±4.1^b^
	E	65.6±8.6^cd^	35.1±3.2^abc^	84.1±0.6^c^	84.0±1.6^c^
Tropical almond	NE	51.5±2.6^a^	29.0±8.1^ab^	46.2±0.5^a^	47.7±9.6^a^
	E	71.4±6.5^de^	38.9±7.3^abc^	84.7±1.4^c^	84.7±1.1^c^
SEM		2.33	1.83	2.49	2.67
p value					
Feedstuff		0.006	0.053	<0.001	0.001
Extraction		<0.001	0.036	<0.001	<0.001
Feedstuff×extraction		0.385	0.509	<0.001	0.001

Different superscripts within the same column are statistically different at P<0.05. IVDMD=*In vitro* dry matter digestibility, IVOMD=*In vitro* organic matter digestibility, NH_3_=Ammonia, SEM=Standard error of mean, VFA=Volatile fatty acid, E=Extracted, NE=Nonextracted

## Discussion

High EE contents of soybean, winged bean, rubber seed, and tropical almond observed in the present study were in agreement with some other reports [7-9]. Winged bean apparently contains CP comparable or even higher than that of soybean. Some studies have reported the high CP content of winged bean. For instance, Kantha and Erdman [18] reported that winged bean contained 29.3-39.0% protein. Other authors also reported that protein content of the bean ranged from 27.8% to 41.9% [19-21]. In agreement with our view, Lepcha *et al*. [22] described in a recent review that winged bean is a valuable alternative protein source to soybean, particularly in the tropics, due to its analog nutritional quality and yield. The CP contents of rubber seed and tropical almond were much lower in comparison to soybean apparently due to their higher EE contents. Other authors confirmed high EE contents of rubber seed and tropical almond. Suprayudi *et al*. [8] and Ladele *et al*. [9] reported that lipid contents of rubber seed and tropical almond were 49.3 and 61.8% DM, respectively; these values were higher in comparison to our results.

Soxhlet extraction using petroleum ether was able to effectively remove lipid from the materials so that their EE contents became <1% DM. Such extraction method is a common method for lipid extraction and has frequently been used for removing lipid from various oilseeds [23,24]. Another method for lipid extraction is mechanical extraction [25], but generally, the method results in a lower lipid recovery in comparison to solvent extraction. In a study of Ali and Watson [26], for instance, maximum oil yield extracted from flax seed was 22.6 and 36.3% using mechanical oil expeller and organic solvent extraction methods, respectively. Other chemical constituents in these materials became more concentrated after lipid removal as shown by the higher CP and CF contents. Such an increase of CP in oilseed is typical after lipid extraction process as also observed by other authors. Full-fat soybean contained 40.8% CP (19.3% EE) and it increased to 50.2% CP (1.6% EE) after solvent extraction using hexane [27]. Similarly, CP content of rubber seed increased from 21.9% to 33.8% DM after an oil extraction procedure [8].

Intact winged bean resulted in the highest *in vitro* gas production among other feed materials due to its comparatively lowest EE content. Conversely, high EE present in intact tropical almond led to its low gas production when incubated in the *in vitro* system. Lipid has been known to contribute to low *in vitro* gas production [28,29]. The component undergoes lipolysis in the rumen to result in fatty acids and glycerol through the action of lipolytic microbes. Main bacteria species responsible for lipolysis is *Anaerovibrio lipolytica* that produces two hydrolytic enzymes, that is, cell-bound esterase and extracellular lipase, which have activity on triglycerides and esterified fatty acids [30]. Fatty acids are not degraded or fermented by rumen microbes, but unsaturated fatty acids are hydrogenated into various fatty acid isomers with higher saturation degree and carried out mainly by *Butyrivibrio fibrisolvens* and *Clostridium proteoclasticum* [30,31]; such hydrogenation process does not generate gas. Although fermentation of glycerol in the rumen produces gas [32], the amount is relatively small [33].

An increase of *in vitro* gas production of all feed materials after lipid removal confirms the negative relationship between lipid and the parameter. Following lipid extraction, the highest gas production of rubber seed meal is apparently related to its lowest CF content than those of other materials. This implies that the type of carbohydrate present in rubber seed meal is mainly non-fiber carbohydrate. Such non-fiber carbohydrate is fermented at a faster rate in comparison to fiber [34] and therefore results in higher gas production. In addition, comparatively low CP content of rubber seed meal contributes to the high *in vitro* gas production since protein has been known to produce less gas as compared to carbohydrate [28]. In agreement with our findings, Maccarana *et al*. [29] observed that a low neutral detergent fiber (NDF) diet produced higher gas production per gram DM incubated than that of a high-NDF diet. The authors also further observed that a high CP diet resulted in lower gas production as compared to a low CP diet.

The pattern of total VFA concentration apparently follows that of gas production since both parameters are end products of microbial fermentation in the rumen. Getachew *et al*. [35] observed that *in vitro* gas production measured at 24 h incubation had a positive correlation (r=0.76; p<0.001) with total VFA concentration; such correlation was based on a total of 38 samples from 12 feedstuffs. In another study, Getachew *et al*. [36] found that *in vitro* gas production calculated from VFA had a good relationship with the measured *in vitro* gas production (R^2^=0.94; p<0.001) and that finding allows accurate prediction of VFA from gas production. High concentration of ammonia in the present experiment is expected since all the feed materials used, both non-extracted and extracted materials, are rich in CP. Feed protein undergoes microbial degradation to result in various peptides and amino acids, and the latter components are deaminated into α-keto acid and ammonia [37]. Therefore, there is a positive correlation between CP content in feed and ruminal ammonia concentration as confirmed by some other studies [35,38].

The IVOMD values of intact soybean and soybean meal obtained in the present experiment were within the normal range of *in vivo* OM digestibility reported in Feedipedia, i.e., 76.3-92% and 86.0-98.0%, respectively [39]. This may indicate the high validity of the data presented. Similar IVDMD and IVOMD values among intact soybean, winged bean, and rubber seed indicate that winged bean and rubber seed can be used as alternatives to soybean in a ruminant diet. Low IVDMD and IVOMD of tropical almond are apparently related to its high EE content as discussed above. After lipid extraction, all these materials are equal with regard to their IVDMD and IVOMD. Thus, in case, there is a shortage supply of soybean meal, defatted winged bean, rubber seed, and/or tropical almond may be used as alternative protein supplements in a ruminant diet.

## Conclusion

Intact winged bean has higher CP content than that of full-fat soybean, but it is not the case for intact rubber seed and tropical almond due to their high EE contents. Lipid extraction improves the nutritional quality of winged bean, rubber seed, and tropical almond and of comparable quality with defatted soybean *in vitro*. Further *in sacco* and *in vivo* evaluations of these alternative protein supplements are required to assess their protein quality and utilization comprehensively.

## Authors’ Contributions

AJ: Design and supervised the experiment, analyzed the data, and drafted the manuscript. RPH and RFR: Executed the experiment and carried out laboratory analysis. N: Supervised the experiment and revised the manuscript. All authors read and approved the final manuscript.

## References

[1] Laconi E.B, Jayanegara A (2015). Improving nutritional quality of cocoa pod (*Theobroma cacao*) through chemical and biological treatments for ruminant feeding *In vitro* and *in vivo* evaluation. Asian Australas. J. Anim. Sci.

[2] Maxin G, Ouellet D.R, Lapierre H (2013). Ruminal degradability of dry matter, crude protein, and amino acids in soybean meal, canola meal, corn, and wheat dried distillers grains. J. Dairy Sci.

[3] Jayanegara A, Palupi E (2010). Condensed tannin effects on nitrogen digestion in ruminants: A meta-analysis from *in vitro* and *in vivo* studies. Media Peternak.

[4] Amanlou H, Farahani T.A, Farsuni N.E (2017). Effects of rumen undegradable protein supplementation on productive performance and indicators of protein and energy metabolism in Holstein fresh cows. J. Dairy Sci.

[5] Vollmann J (2016). Soybean versus other food grain legumes: A critical appraisal of the United Nations international year of pulses 2016. Bodenkult. J. Land Manag. Food Environ.

[6] Kovalenko I.V, Rippke G.R, Hurburgh C.R (2006). Determination of amino acid composition of soybeans (*Glycine max*) by near-infrared spectroscopy. J. Agric. Food Chem.

[7] Makeri M.U, Abdulmannan F, Ilowefah M.A, Chiemela C, Bala S.M, Muhammad K (2017). Comparative physicochemical, functional and structural characteristics of winged bean (*Psophocarpus tetragonolobus* DC) and soybean (*Glycine max*) protein isolates. J. Food Meas. Charact.

[8] Suprayudi M.A, Inara C, Ekasari J, Priyoutomo N, Haga Y, Takeuchi T, Satoh S (2015). Preliminary nutritional evaluation of rubber seed and defatted rubber seed meals as plant protein sources for common carp *Cyprinus carpio* L. juvenile diet. Aquac. Res.

[9] Ladele B, Kpoviessi S, Ahissou H, Gbenou J, Kpadonou-Kpoviessi B, Ladekan E.Y, Kpoviessi D.S, Gbaguidi F, Yehouenou B, Quetin-Leclercq J, Figueredo G, Moudachirou M, Accrombessi G.C (2016). Chemical composition and nutritional properties of *Terminalia catappa* L. oil and kernels from Benin. Comptes Rendus Chim.

[10] Duarte A.C, Durmic Z, Vercoe P.E, Chaves A.V (2017). Dose-response effects of dietary pequi oil on fermentation characteristics and microbial population using a rumen simulation technique (Rusitec). Anaerobe.

[11] Górka P, Castillo-Lopez E, Joy F, Chibisa G.E, McKinnon J.J, Penner G.B (2015). Effect of including high-lipid by-product pellets in substitution for barley grain and canola meal in finishing diets for beef cattle on ruminal fermentation and nutrient digestibility. J. Anim. Sci.

[12] Zanetti F, Monti A, Berti M.T (2013). Challenges and opportunities for new industrial oilseed crops in EU-27: A review. Ind. Crops Prod.

[13] Savadi S, Lambani N, Kashyap P.L, Bisht D.S (2017). Genetic engineering approaches to enhance oil content in oilseed crops. Plant Growth Regul.

[14] AOAC (2005). Official Methods of Analysis of AOAC International.

[15] Theodorou M.K, Williams B.A, Dhanoa M.S, McAllan A.B, France J (1994). A simple gas production method using a pressure transducer to determine the fermentation kinetics of ruminant feeds. Anim. Feed Sci. Technol.

[16] Tilley J.M.A, Terry R.A (1963). A two-stage technique for the *in vitro* digestion of forage crops. Grass Forage Sci.

[17] Jayanegara A, Dewi S.P, Laylli N, Laconi E.B, Nahrowi N, Ridla M (2016). Determination of cell wall protein from selected feedstuffs and its relationship with ruminal protein digestibility *in vitro*. Media Peternak.

[18] Kantha S.S, Erdman J.W (1984). The winged bean as an oil and protein source: A review. J. Am. Oil Chem. Soc.

[19] Ibuki F, Kotaru M, Kan K.K, Ikeuchi T, Kanamori M (1983). Chemical composition of winged bean (*Psophocarpus tetragonolobus*) varieties. J. Nutr. Sci. Vitaminol.

[20] Gross R (1983). Composition and protein quality of winged bean (*Psophocarpus tetragonolobus*). Plant Foods Hum. Nutr.

[21] Prakash A, Niranjan S.K, Te D (2001). Underutilised legumes: Potential sources for low-cost protein. Int. J. Food Sci. Nutr.

[22] Lepcha P, Egan A.N, Doyle J.J, Sathyanarayana N (2017). A review on current status and future prospects of winged bean (*Psophocarpus tetragonolobus*) in tropical agriculture. Plant Foods Hum. Nutr.

[23] Bouallegue K, Allaf T, Ben Y.R, Allaf K (2016). Texturing and instant cooling of rapeseed as pretreatment prior to pressing and solvent extraction of oil. Food Bioprocess Technol.

[24] Castejón N, Luna P, Señoráns F.J (2018). Alternative oil extraction methods from *Echium plantagineum* L. seeds using advanced techniques and green solvents. Food Chem.

[25] Savoire R, Lanoisellé J.L, Vorobiev E (2013). Mechanical continuous oil expression from oilseeds: A review. Food Bioprocess Technol.

[26] Ali M, Watson I.A (2014). Comparison of oil extraction methods, energy analysis and biodiesel production from flax seeds: Microwave, ultrasonic and solvent extraction methods for flax seeds. Int. J. Energy Res.

[27] Tacon A.G.J, Haaster J.V, Featherstone P.B, Kerr K, Jackson A.J (1983). Studies on the utilization of full-fat soybean and solvent extracted soybean meal in a complete diet for rainbow trout. Nippon Suisan Gakkaishi.

[28] Getachew G, Blümmel M, Makkar H.P.S, Becker K (1998). *In vitro* gas measuring techniques for assessment of nutritional quality of feeds: A review. Anim. Feed Sci. Technol.

[29] Maccarana L, Cattani M, Tagliapietra F, Bailoni L, Schiavon S (2016). Influence of main dietary chemical constituents on the *in vitro* gas and methane production in diets for dairy cows. J. Anim. Sci. Biotechnol.

[30] Jenkins T.C, Wallace R.J, Moate P.J, Mosley E.E (2007). Recent advances in biohydrogenation of unsaturated fatty acids within the rumen microbial ecosystem. J. Anim. Sci.

[31] Lourenço M, Ramos-Morales E, Wallace R.J (2010). The role of microbes in rumen lipolysis and biohydrogenation and their manipulation. Animal.

[32] Syahniar T.M, Ridla M, Samsudin A.A, Jayanegara A (2016). Glycerol as an energy source for ruminants: A meta-analysis of *in vitro* experiments. Media Peternak.

[33] Avila-Stagno J, Chaves A.V, Ribeiro G, Ungerfeld E.M, McAllister T.A (2014). Inclusion of glycerol in forage diets increases methane production in a rumen simulation technique system. Br. J. Nutr.

[34] Li F, Yang X.J, Cao Y.C, Li S.X, Yao J.H, Li Z.J, Sun F.F (2014). Effects of dietary effective fiber to rumen degradable starch ratios on the risk of sub-acute ruminal acidosis and rumen content fatty acids composition in dairy goat. Anim. Feed Sci. Technol.

[35] Getachew G, Robinson P, DePeters E, Taylor S (2004). Relationships between chemical composition, dry matter degradation and *in vitro* gas production of several ruminant feeds. Anim. Feed Sci. Technol.

[36] Getachew G, Makkar H.P.S, Becker K (2002). Tropical browses: Contents of phenolic compounds *in vitro* gas production and stoichiometric relationship between short chain fatty acid and *in vitro* gas production. J. Agric. Sci.

[37] Bach A, Calsamiglia S, Stern M.D (2005). Nitrogen metabolism in the rumen. J. Dairy Sci.

[38] Jayanegara A, Wina E, Soliva C.R, Marquardt S, Kreuzer M, Leiber F (2011). Dependence of forage quality and methanogenic potential of tropical plants on their phenolic fractions as determined by principal component analysis. Anim. Feed Sci. Technol.

[39] Food and Agriculture Organization of the United Nations (2018). Feedipedia–Animal Feed Resources Information System.

